# Furfural induces reactive oxygen species accumulation and cellular damage in *Saccharomyces cerevisiae*

**DOI:** 10.1186/1754-6834-3-2

**Published:** 2010-01-15

**Authors:** Sandra A Allen, William Clark, J Michael McCaffery, Zhen Cai, Alison Lanctot, Patricia J Slininger, Z Lewis Liu, Steven W Gorsich

**Affiliations:** 1 Biology Department, Central Michigan University, Mt Pleasant, MI 48859, USA; 2 Integrated Imaging Center, Department of Biology, Johns Hopkins University, Baltimore, MD 21218, USA; 3 National Center for Agricultural Utilization Research, Agricultural Research Service, United States Department of Agriculture, Peoria, IL 61604, USA

## Abstract

**Background:**

Biofuels offer a viable alternative to petroleum-based fuel. However, current methods are not sufficient and the technology required in order to use lignocellulosic biomass as a fermentation substrate faces several challenges. One challenge is the need for a robust fermentative microorganism that can tolerate the inhibitors present during lignocellulosic fermentation. These inhibitors include the furan aldehyde, furfural, which is released as a byproduct of pentose dehydration during the weak acid pretreatment of lignocellulose. In order to survive in the presence of furfural, yeast cells need not only to reduce furfural to the less toxic furan methanol, but also to protect themselves and repair any damage caused by the furfural. Since furfural tolerance in yeast requires a functional pentose phosphate pathway (PPP), and the PPP is associated with reactive oxygen species (ROS) tolerance, we decided to investigate whether or not furfural induces ROS and its related cellular damage in yeast.

**Results:**

We demonstrated that furfural induces the accumulation of ROS in *Saccharomyces cerevisiae*. In addition, furfural was shown to cause cellular damage that is consistent with ROS accumulation in cells which includes damage to mitochondria and vacuole membranes, the actin cytoskeleton and nuclear chromatin. The furfural-induced damage is less severe when yeast are grown in a furfural concentration (25 m*M*) that allows for eventual growth after an extended lag compared to a concentration of furfural (50 m*M*) that prevents growth.

**Conclusion:**

These data suggest that when yeast cells encounter the inhibitor furfural, they not only need to reduce furfural into furan methanol but also to protect themselves from the cellular effects of furfural and repair any damage caused. The reduced cellular damage seen at 25 m*M *furfural compared to 50 m*M *furfural may be linked to the observation that at 25 m*M *furfural yeast were able to exit the furfural-induced lag phase and resume growth. Understanding the cellular effects of furfural will help direct future strain development to engineer strains capable of tolerating or remediating ROS and the effects of ROS.

## Background

The continued use of fossil fuels has raised environmental, economical and political concerns and, as a result, research into improving alternative and renewable energy strategies is of great importance. Bioethanol is one such alternative energy source. Most bioethanol produced today takes advantage of ethanologenic microorganisms fermenting agricultural products such as cornstarch or sugar cane. Starch and sugar cane sources are currently being used to produce competitively priced ethanol in countries such as Brazil, Canada and the USA. Unfortunately, these sources are not sufficient to supply the world bioenergy needs due to the role they play in human and livestock consumption [1]. Thus, the goal of having a bioethanol fuel economy must include in its vision the use of lignocellulosic-biomass waste from agriculture, forests, industry and the municipalities. Current technologies make the use of lignocellulosic-biomass inefficient. However, programs using agricultural and softwood biomass are currently producing ethanol in Sweden, the USA and Canada, with the later having established a committed plant for the production of bioethanol from lignocellulose [2-4].

In order to release fermentable sugars from lignocellulosic biomass, a weak acid pre-treatment step is often employed. However, this process generates fermentation inhibitors, which include aldehydes (furan aldehydes), ketones, phenolics and organic acids [5-9]. Two furan aldehydes are 2-furaldehyde (furfural) and 5-hydroxymethylfurfural (HMF), which are degradation products of xylose and glucose, respectively. In order to protect themselves yeast reduce these furan aldehydes to their less toxic alcohol derivatives, furan methanol and furan dimethanol, in NAD(P)H-dependent reactions. This conversion occurs during the growth lag phase when ethanol production and many enzymes are inhibited [5,10,11]. Once these inhibitors are reduced, growth resumes. In addition to detoxifying the furan aldehydes, yeast cells must survive the toxic effects and repair any damages caused by them. However, little is known about the toxic effects of furan aldehydes on cells.

The NADPH producing pentose phosphate pathway (PPP) plays an essential role in furfural tolerance [12]. When single PPP genes (*ZWF1*, *GND1*, *TKL1 *or *RPE1*) are absent, yeast, that would normally allow growth after a 24 hour lag, are unable to grow when concentrations of furfural (25 m*M*) are present [13]. The greatest growth defect is seen when the *ZWF1 *gene is disrupted. *ZWF1 *encodes glucose-6-phosphate dehydrogenase, which catalyzes the rate-limiting step of the PPP and produces NADPH. This growth defect is probably not due to an inability to reduce furfural, as furfural can be reduced using NADH. However, the PPP's NADPH is also an important co-factor used to protect cells against cellular stress caused by reactive oxygen species (ROS).

ROS are generated in cells as metabolic byproducts, the accumulation of which can be increased by environmental conditions, genetic mutations and cell ageing [14-16]. ROS include hydrogen peroxide (H_2_O_2_), superoxide anion (O_2 _^-^), and the hydroxyl radical (OH^-^). ROS are known to damage DNA, proteins, lipids and the cytoskeleton and to induce programmed cell death [17-19]. Cells can protect themselves from ROS by activating certain genes, such as the PPP's *ZWF1*, which also is an essential gene for furfural tolerance. Yeast lacking the rate limiting PPP gene, *ZWF1*, have an increased sensitivity to ROS [20]. The role of the PPP in ROS protection is likely due to the NADPH that it produces [21]. The reducing power of NADPH is used by many stress protection enzymes, such as those encoded by *OAR1*, *OYE2*, *TSA1 *and *GLR1*, which encode the enzymes mitochondrial 3-oxoacyl- [acyl-carrier-protein] reductase, old yellow enzyme, thioredoxin peroxidase and glutathione oxioreductase, respectively [22-24].

Since the PPP is necessary to protect cells against ROS and furfural, we proposed that furfural's role in cellular toxicity involves ROS related damage. In this study, we demonstrate that furfural causes an accumulation of ROS and cellular damage to mitochondria, vacuoles, actin and nuclear chromatin when healthy and exponentially growing cells are transferred to furfural. The damage is less severe in concentrations of furfural (25 m*M*) that normally allow growth after a 24 hour lag as opposed to concentrations of furfural (50 m*M*) that prevent growth completely (Additional file 1). This reduced degree of cellular damage may be indicative of why yeast can survive at lower concentrations of furfural. Moreover, these data will be useful in the development of more robust yeast strains.

## Results and discussion

### Accumulation of reactive oxygen species in cells

**Figure 1:**
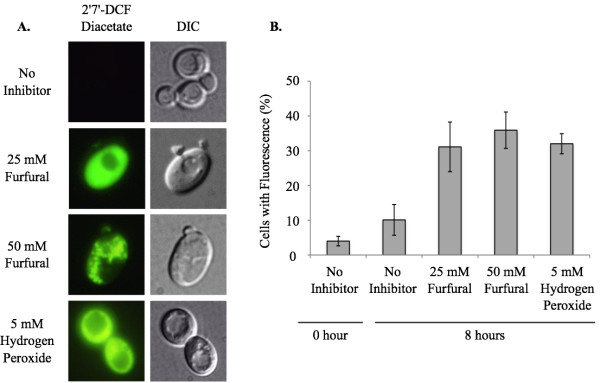
**Furfural induces the accumulation of reactive oxygen species (ROS)**. Exponentially growing yeast cells were treated with no inhibitor, 25 m*M *furfural, 50 m*M *furfural or 5 m*M *hydrogen peroxide (positive control for ROS). (A) Representative images of cells stained with the ROS indicator dye 2'7' DCF-diacetate (left column) and differential interference contrast (DIC) (right column) are shown. Images of cells were at the 8 h time point. (B) Percent of yeast cells that stained positive for ROS by 2'7' DCF-diacetate at 0 h and 8 h. Data represent an average of five experiments with standard error indicated. At each time point at least 100 cells were examined.

Yeast in exponential growth was transferred to media containing no inhibitor, 25 or 50 m*M *furfural or 5 m*M *hydrogen peroxide. Furfural addition immediately sent the healthy cells into a growth lag phase even though there were sufficient nutrients available (Additional file 1). Hydrogen peroxide present in the medium served as a positive control of ROS. Cell cultures were allowed to grow at 25°C and aliquots of cells were removed and stained with the ROS detecting dye, 2'7'- DCF diacetate (Figure 1). Cells staining positive for ROS were counted in order to determine the percent of cells containing accumulated ROS. For each sample at least 100 cells were examined. At 0 h 4% of the cells had a positive ROS signal. After 8 h of growth 10% of the cells had a positive ROS signal with no furfural present. At the same time point, 31% and 36% of cells stained positive when 25 and 50 m*M *furfural were present, respectively. This was consistent with the 32% of cells exposed to 5 m*M *hydrogen peroxide, which is a known inducer of ROS (Figure 1). Interestingly, cells exposed to 50 m*M *furfural had an aggregated staining pattern, which is strikingly different from the even distribution of fluorescence seen when cells are exposed to 25 m*M *furfural or 5 m*M *hydrogen peroxide. We speculate these aggregates are either aggregated proteins or membranes damaged by furfural. This extreme staining difference may be a result of the stronger growth inhibition of furfural at 50 m*M *[13] (Additional file 1).

### Cellular damages determined by transmission electron microscopy (TEM) analysis

**Figure 2:**
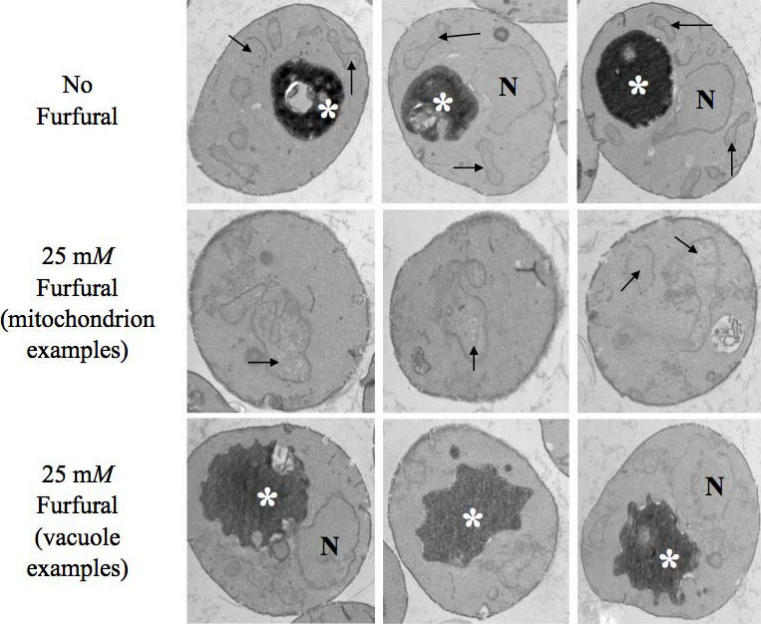
**Furfural causes internal cellular damage**. Exponentially growing yeast cells grown with either no inhibitor (1st row) or 25 m*M *furfural (2nd and 3rd rows) were fixed and thin-sectioned for transmission electron microscopy analysis after they had been exposed to furfural for 8 h. Mitochondria are indicated by arrows, vacuoles by asterisks, and nuclei by N.

ROS are known to damage DNA, proteins, lipids, and the cytoskeleton [17-19]. We used TEM to test whether furfural could induce similar internal cellular damage (Figure 2). Fixed yeast cells, either exposed to 25 m*M *furfural or no inhibitors, were processed for thin-section TEM analysis. When no inhibitor was present, mitochondrial structures were broadly distributed around the cell periphery with a typical morphology and contained internal cristae. In addition, vacuolar structures were also typical, appearing as single dark structures with smooth edges. In the presence of 25 m*M *furfural, mitochondria appeared highly aggregated and swollen with less structured cristae and were clustered towards the cell interior. Though the vacuoles were about the same size as the untreated cells, their edges were not smooth, but rather lobular. Interestingly, the furfural treated cells contained a lightly stained background as opposed to the untreated cells that had a clear background. We were unable to identify what this was but we suspected that it was damaged cytoskeleton or aggregated proteins; both would be consistent with ROS [18,19]. The nuclear membrane in furfural treated and untreated cells appeared unaffected (Figure 2). Interestingly, the yeast external cell wall did not appear to be affected by either 25 or 50 m*M *furfural as observed by scanning electron microscopy (data not shown). This suggests that, prior to entering the cell, furfural does not damage the cell wall.

### Mitochondrial membrane damage

**Figure 3:**
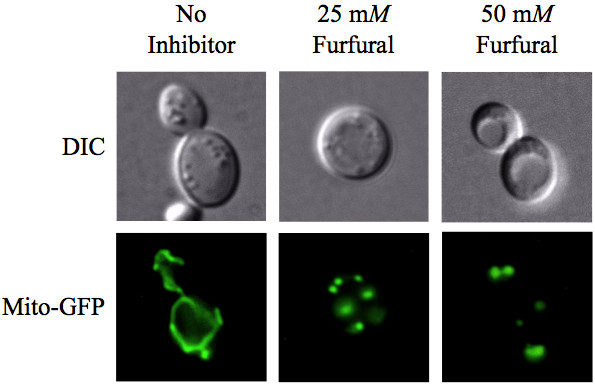
**Furfural causes mitochondrial membrane morphology to go from tubules to aggregates**. Exponentially growing yeast cells expressing mitochondrial targeted green fluorescent protein were untreated or treated with 25 m*M *or 50 m*M *furfural. Representative images of yeast with no inhibitor (left column; tubular), exposed to 25 m*M *furfural (middle column; evenly distributed fragments) or 50 m*M *furfural (right column; aggregated) are shown. Images of cells were taken 6 h after furfural treatment.

Yeast (SGY229) cells in exponential growth were either not treated or treated with 25 m*M *or 50 m*M *furfural. Cells containing a mitochondrial targeted green fluorescent protein (pVT100U-mtGFP) allowed for visualization of mitochondria, which usually appear as a tubular network of membranes localized to the cells cortex [25]. At 0 h, cells contained the typical tubular shaped mitochondria in 87% of observed cells (Figure 3, Table 1). Mitochondria remained tubular in 80% of the untreated cells at 6 h. However, in the presence of 25 or 50 m*M *furfural mitochondria either fragmented evenly (41% and 45%, respectively) or aggregated to one side of the cell (9% and 45%, respectively). In the untreated cells mitochondria remained tubular until 48 h when 79% of cells contained evenly distributed fragments, which is typical of mitochondria in cells going from exponential to stationary growth phase. Cultures treated with 25 m*M *furfural at 24 h and 48 h contained mitochondria that were predominately fragmented in 49% and 53% of the cells. Yeast cultures treated with 50 m*M *furfural continued to display predominantly aggregated mitochondria that were not evenly distributed in 66% of the cells at 24 h and 100% of the cells at 48 h. Fragmented and aggregated mitochondria are phenotypes associated with some yeast mutants like *mgm1*. These mutants lose their mitochondrial DNA, making them respiratory incompetent, and they exhibit poor growth when dextrose is their carbon source and fail to grow when glycerol is their carbon source [26,27]. In addition, this mutant and its homologue mutants in *Drosophilae*, *Caenorhabditis elegans*, and humans are more sensitive to ROS [28-30].

**Table 1:** Mean count of yeast cells expressing mitochondrial targeted green fluorescent protein indicating mitochondrial membrane damages from tubular to aggregated morphologies caused by furfural treatment.

Hour	Category*	Control	25 mM Furfural	50 mM Furfural
6	Tubular	80 ± 13	50 ± 5	10 ± 2
6	Fragmented	19 ± 13	41 ± 2	45 ± 3
6	Aggregated	1 ± 0.4	9 ± 2	45 ± 2
24	Tubular	94 ± 4	27 ± 8	4 ± 4
24	Fragmented	6 ± 2	49 ± 4	33 ± 23
24	Aggregated	< 1 ± 1	24 ± 10	66 ± 28
48	Tubular	17 ± 5	16 ± 10	0
48	Fragmented	79 ± 3	53 ± 15	0
48	Aggregated	4 ± 2	31 ± 6	100 ± 0

Prior to adding furfural, 87%, 12% and 1% of cells contained tubular, fragmented and aggregated mitochondria, respectively. Data represent averages of three experiments with standard error indicated. At least 100 cells were examined at each time point.

* Tubular mitochondria are a network of evenly distributed tubules that are often connected; fragmented mitochondria are small evenly distributed spherical structures; aggregated mitochondria are small and clustered spherical structures that are not evenly distributed.

Our observation that 25 m*M *furfural caused a less severe phenotype compared to 50 m*M *furfural is consistent with 25 m*M *furfural treated cells being able to grow after a 24 h growth lag (Additional file 1). These data are also consistent with our TEM images of mitochondrial clusters (Figure 2).

### Vacuole membrane damage

**Figure 4:**
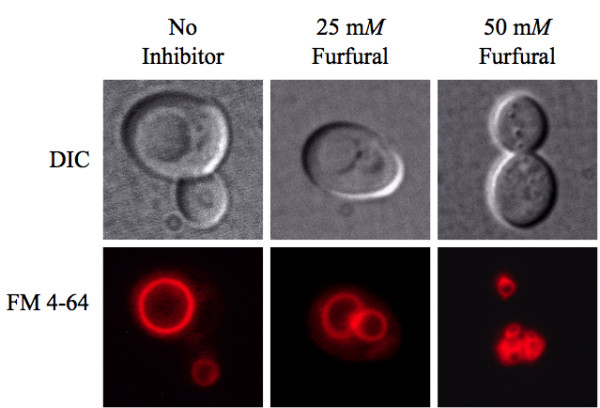
**Furfural causes vacuoles to go from large single organelles to several smaller ones**. Exponentially growing yeast cells were either untreated or treated with 25 m*M *or 50 m*M *furfural. Aliquots of cells were removed and stained with the vacuole targeted dye FM 4-64. Representative images of yeast with no inhibitor (left column; single large vacuoles), exposed to 25 m*M *furfural (middle column; two to four medium-sized vacuoles) and 50 m*M *furfural (right column; small and fragmented) are shown. Images of cells were taken 6 h after furfural treatment.

Yeast in exponential growth were either not treated or treated with 25 or 50 m*M *furfural. At each time point aliquots of cells were stained with the vacuole dye FM 4-64^® ^(Figure 4, Table 2). At 0 h, 96% of the cells contained single and large normal vacuoles, which is the typical appearance of the yeast vacuole [31]. When no inhibitor was present vacuoles continued to predominately be a single and large structure through 48 h. When 25 or 50 m*M *furfural was added, vacuoles fragmented into two to four medium sized vacuoles or greater than four smaller vacuoles, respectively. As with mitochondria, the vacuoles fragmented and aggregated more in the presence of 50 m*M *furfural. Our TEM images did not show fragmented vacuoles. However, we suspect the lobular shape of the vacuoles in the TEM images were showing small vacuoles that were clustered together to produce the lobular affect (Figure 2). The effect of furfural-induced vacuole fragmentation is not clear, but it is known that drugs such as nocodazole cause vacuoles to fragment, probably associated with this drugs affect on microtubules [32]. Interestingly, vacuole fragmentation is also seen with some mutants such as *vac8*, which also affects cellular endocytosis [33,34]. Whether or not furfural causes a block in endocytosis or if blocking endocytosis helps protect yeast is not known.

**Table 2:** Mean count of yeast cells displaying vacuole damages from a single large to numerous small sized vacuoles caused by furfural treatment.

Hour	Category	Control	25 mM Furfural	50 mM Furfural
6	Single/large	86.5 ± 6	48 ± 8	30.0 ± 3
	2-4/Medium	8.5 ± 1	22 ± 4	35.5 ± 4
	> 4/Small	5.0 ± 5	30 ± 12	34.5 ± 6
24	Single/large	77 ± 13	36 ± 16	7.5 ± 8
	2-4/Medium	16 ± 6	21 ± 0.2	18.0 ± 13
	> 4/Small	7 ± 8	43 ± 16	74.5 ± 21
48	Single/large	79.5 ± 1	28 ± 5	1 ± 1
	2-4/Medium	15.5 ± 4	22 ± 5	7 ± 3
	> 4/Small	5.0 ± 3	49 ± 7	92 ± 4

Prior to adding furfural, 96%, 2% and 2% of cells contained large, medium and small shaped vacuoles, respectively. Data represent averages of two experiments with standard error indicated. At least 100 cells were examined at each time point.

### Nuclear chromatin disorganization

**Figure 5:**
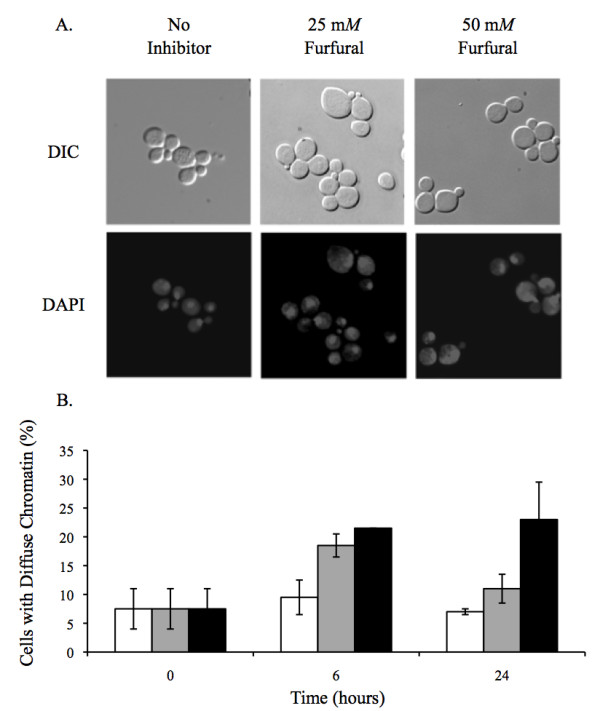
**Furfural causes nuclear chromatin to go from tight organized spheres to diffuse unorganized structures**. Exponentially growing yeast cells were either untreated or treated with 25 m*M *or 50 m*M *furfural. Aliquots of cells were removed and stained with the DNA specific dye DAPI, which is shown as lightly stained structures in the cytoplasm. (A) Representative images observed with no inhibitor (left column; tightly compacted spheres), 25 m*M *furfural (middle column; diffuse chromatin) or 50 m*M *furfural (right column; diffuse chromatin) are shown. Stained chromatin appear as white structures. Images of cells were taken 6 h after furfural treatment. (B) Percent of cells at each concentration of furfural that contain diffuse chromatin similar to right two images in (A) at 0 h, 6 h and 24 h. Data represent an average of three experiments with standard error indicated. At each time point 100 or more cells were examined.

TEM analysis did not reveal any obvious nuclear external damage (Figure 2). However, we were interested in whether or not the nuclear chromatin inside the nucleus was damaged. Yeast nuclei exposed to ROS are known to become less compacted and appear larger and more diffuse when stained with the DNA specific dye, DAPI [35,36]. In order to test this in our furfural treated cells we grew cells to exponential phase and treated them with 25 or 50 m*M *furfural or with no inhibitor. At various time points aliquots of cells were removed and stained with DAPI. At 0 h, 7.5% of the cells contained abnormal diffuse nuclear chromatin while the remaining cells contained chromatin that appeared as normal tightly compacted spheres. Upon adding 25 or 50 m*M *furfural the nuclear chromatin became disorganized and diffuse in 18.5% and 21.5% of the cells at 6 h, respectively, and 11% and 23% of cells at 24 h, respectively (Figure 5). Our DAPI observations are consistent with cells undergoing ROS induced stress [35,36]. It is interesting that by 24 h our data suggests that the nuclear chromatin damage is recovering faster than the mitochondrion and vacuole membrane damage (Figures 3 and 4). This difference in the time required to repair each substrate (membrane versus chromatin) could be linked to how fast the cell is capable of repairing different cell components, the degree of damage to each cell component or, possibly, the cell recognizes the importance of repairing the DNA containing chromatin. Together, our TEM and DAPI data suggest that furfural does cause chromatin damage without causing obvious nuclear structural damage.

### Actin cytoskeleton damage

**Figure 6:**
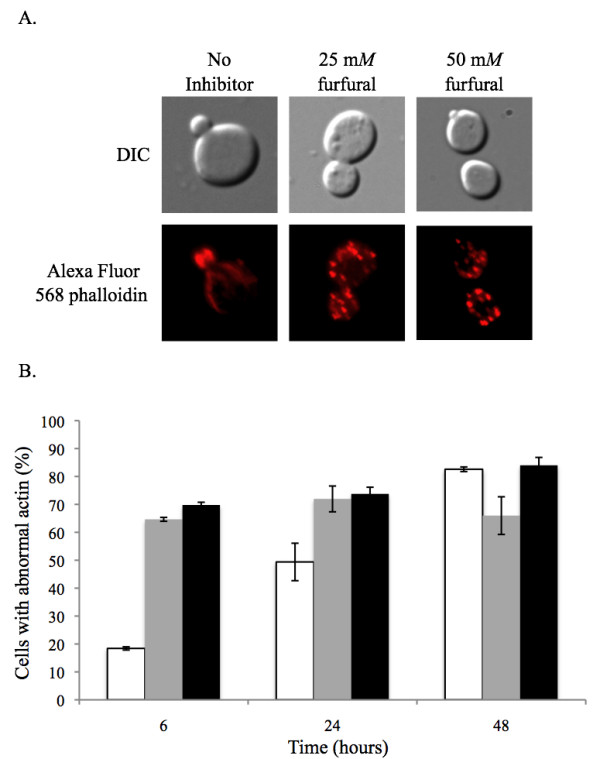
**Furfural causes the actin cytoskeleton to go from predominantly cables to patches**. Exponentially growing yeast cells were either untreated or treated with 25 m*M *or 50 m*M *furfural. (A) Representative images observed with no inhibitor (left column; actin cables in mother cell), 25 m*M *furfural (middle column; actin patches in mother cell) or 50 m*M *furfural (right column; actin patches in mother cell) are shown. Images of cells were taken at the 6 h time point. (B) Percent of cells at each concentration of furfural at 0 h, 6 h and 24 h that contain only actin patches similar to right two images in (A). Data represent an average of two experiments with standard error indicated. At each time point 100 cells were examined.

The actin cytoskeleton in a healthy growing yeast cell will contain long thin actin cables in the mother cell that extend into the daughter bud where they end as an actin patch [37]. TEM analysis did not provide clear evidence of any actin structure damage induced by furfural (Figure 2). However, since actin damage is seen in ROS induced apoptosis [38], we investigated the actin structures in cells exposed to furfural. Yeast in exponential growth were either not treated or treated with 25 or 50 m*M *furfural. At various time points aliquots of cells were removed and their actin cytoskeleton stained with Alexa Fluor^® ^568 phalloidin. At 0 h, 67% of the cells contained actin structures consistent with normal growth (actin cables in the mother cell and patches in the daughter bud) and 33% lacked cables and only had patches (Figure 6). In cell cultures without inhibitor normal actin structures remained in most of the cells up until 24 h. By 48 h many of these cells contained abnormal actin, which is consistent with our previous observations of cultures at stationary growth phase (data not shown). Cultures containing 25 or 50 m*M *furfural contained predominantly abnormal actin structures (over 70% with only actin patches) through 48 h. The 25 m*M *treated cells contained less abnormal actin compared to the 50 m*M *treated cells by 48 h. However, the amount of abnormal actin from 6 h to 48 h in the 25 m*M *treated cells does not change significantly. Mitochondria and vacuole membrane damage (Tables 1 and 2) appears to recover faster than actin damage. The significance of this observation is unclear. Perhaps, since actin cables are needed for cell budding, yeast cells do not expend energy making actin cables until other components are repaired and the cell is ready to bud. Alternatively, actin patches are known to play a role in endocytosis. Whether or not endocytosis is important in furfural tolerance is not known. However, it is noteworthy that the gene, *BRE4*, has been linked to both endocytosis and furfural tolerance [13,39]. This link needs to be further investigated.

## Conclusion

Prior to this study it was not known if furfural causes oxidative or internal cellular damage. The lignocellulose-derived inhibitor, furfural, prevents yeast cells from growing and producing ethanol until furfural is reduced to furan methanol by NAD(P)H-dependent reactions. However, what was happening to these yeast cells while furfural was being reduced was not known. We show that furfural does cause an accumulation of reactive oxygen species (ROS) (Figure 1) and damages mitochondrial and vacuole membranes (Figures 2, 3, 4), nuclear chromatin (Figure 5) and the actin cytoskeleton (Figure 6). This is consistent with known targets of ROS [17-19]. Surprisingly, preliminary results indicate that furfural-induced ROS did not cause programmed cell death (data not shown), which is often the final consequence of ROS [35,36]. These programmed cell death experiments are being further investigated. Moreover, we suspect that the damage to the cell is only present inside the cell, as no obvious damage to the external cell wall was detected by scanning electron microscope (SEM) analysis (data not shown). Alternatively, furfural may damage the external side of the cell, but it remains undetected by the current SEM assay.

Developing future furfural tolerant yeast strains will probably involve one of two strategies. The first is to engineer strains for the improved conversion of furfural to furan methanol. A potential target gene for improved conversion is *YGL157W*, which encodes an aldedyde reductase [40]. A similar strategy proved successful when Petersson et al. (2006) overexpressed *ADH6 *(alcohol dehydrogenase) to improve HMF reduction [41]. The second strategy is to develop a strain that is able to tolerate or remediate ROS and ROS-induced cellular damage more effectively. Potential target genes to engineer for an improved robustness include genes known to function in stress tolerance such as *OAR1*, *TSA1 *and *GLR1 *[22-24]. In order to achieve maximal furfural tolerance it is probable that both increased furfural conversion and ROS tolerance will need to be considered in future strain development strategies.

## Methods

### Yeast growth conditions and reagents

The budding yeast, *S. cerevisiae*, were grown using standard laboratory conditions [42,43]. All standard chemicals were purchased from Sigma-Aldrich (MO, USA). For all experiments, strains derived from FY10 were used [44]. These include BY4741 (SGY110) (MATa his3Δ1 leu2Δ0 met15Δ0 ura3Δ0) and SGY229 (MATa his3Δ1 leu2Δ0 met15Δ0 ura3Δ0+ pVT100U-mtGFP). BY4741 is a standard wild-type lab FY strain (S288C) purchased from Open Biosystems (AL, USA). SGY229 is BY4741 that contains plasmid pVT100U-mtGFP. The plasmid pVT100U-mtGFP contains the *URA3 *gene as an auxotrophic marker and encodes a mitochondrial targeted GFP protein, which enables mitochondrial visualizations [45]. Yeast were grown at 25°C in 3 ml of medium with minimal shaking in 15 ml screw-capped plastic centrifuge tubes (VWR 89039-664) in order to provide a less aerobic environment that better mimics industrial ethanol fermentation. For all experiments, except the mitochondrial studies, yeast was grown in liquid yeast extract-peptone-dextrose (YPD; 2% Bacto peptone; 2% dextrose; 1% yeast extract; pH 5.5). In mitochondria observation experiments yeast were grown in either synthetic defined (SD)-complete or SD-URA (synthetic medium composed of 0.67% yeast nitrogen base, 2% dextrose, and supplemented with nucleic acids and amino acids; pH 5.5) at 25°C. Yeast transformation was performed using a standard lithium acetate transformation protocol [46]. Exponentially growing yeast were treated with 0 m*M*, 25 m*M *or 50 m*M *fresh furfural (Sigma-Aldrich 185914) stored under nitrogen. For the ROS experiments, 5 m*M *hydrogen peroxide (Sigma-Aldrich H1009) was added to exponentially growing yeast. This served as a positive control for ROS [47]. Aliquots of cells were removed for analysis at various time points from 0 h to 48 h.

### Electron microscopy

For SEM yeast were put through an ethanol dehydration series. The samples were left in each step for 10 min at concentrations of 30%, 50%, 70%, 95% and three changes of 100% ethanol. Cells were collected onto membrane filters with 0.22 micron holes, critical point dried from CO_2 _with three different 10-min soaks and 2 min purges and transferred onto stubs with carbon tape. Cells were then analysed using a JEOL 840A SEM. TEM was performed as described in Rieder *et al*. using a JOEL 12 EX TEM [48].

### Fluorescence microscopy and cellular analysis

All fluorescence microscopy was performed using either a Nikon 80i eclipse fluorescent light microscope or an Olympus Fluoview 300 confocal microscope. Depending upon the assay, one of the following three fluorescent filters was used: FITC HYQ fluorescence filter (460-500 nm); TX RED HYQ fluorescent filter (532-587 nm); and ultraviolet filter (325-375 nm).

ROS were measured by adding 10 μg of 2' 7'-dichlorofluorescein diacetate (DCF) (Sigma-35845) (using a 2.5 mg/ml stock in ethanol) to 10^7 ^cells and incubated at 30°C for 2 h. Cells were washed with 1 ml of distilled water and resuspended in 0.1 ml phosphate buffered saline (PBS) pH 7.0 [36]. Cells were observed using the FITC HYQ filter. For each time point at least 100 cells were examined.

In order to view the mitochondrial membranes we transformed pVT100U-mtGFP into BY4741 yeast. Transformants with this plasmid express a mitochondrial targeted GFP (pVT100U-mtGFP) [45] that allows the direct visualization of mitochondria in living cells. Mitochondria were visualized using a FITC HYQ fluorescence filter (460-500 nm). Mitochondria were classified in one of three categories: tubular, fragmented or aggregated. Tubular mitochondria appear as a network of evenly distributed tubules that are often connected. Fragmented mitochondria appear as small spherical structures that are evenly distributed throughout the cell. Aggregated mitochondria appear as small spherical structures that are clustered together and are not evenly distributed in the cell. For each time point at least 100 cells were examined.

Vacuole morphology was visualized by taking 10^7 ^cells and resuspending them in 250 μl YPD + 80 m*M *FM 4-64^® ^in dimethyl sulphoxide (Invitrogen-Molecular Probes T13320, CA, USA). FM 4-64 is a lipophilic vital stain that becomes internalized and collects in vacuoles. Cells were incubated at 30°C for 30-60 min, collected and resuspended in 5 ml of YPD in a shake flask and incubated at 30°C for 90-120 min. Cells were collected and washed once with 5 ml of sterile deionized water. They were then resuspended in 25 ml yeast nitrogen base (0.67% ; pH 5.5). Vacuole stained cells were visualized using the TX RED HYQ filter (532-587 nm) [49]. For each time point at least 100 cells were examined.

Nuclear chromatin was visualized by taking 0.2 ODs (A_600_; 1 cm) of cells and washing them one time with deionized water. Cells were fixed by resuspending them in 10 μl of deionized water and 190 μl of 100% ethanol. One μl of a 2 mg/ml diaminophenylindole (DAPI; Roche 10236276001, CA, USA) solution in deionized water was added to the fixed cells and gently mixed. Cells were then immediately collected and washed three times with 200 μl deionized water. In the final wash cells were resuspended in 50 μl of deionized water. Nuclear chromatin was visualized using a UV-2E/C filter (325-375 nm) [50]. Nuclear chromatin was classified as either a tightly compacted sphere that covered a small part of the cell or as a diffuse structure that covered a large part of the cell. For each time point at least 100 cells were examined.

The actin cytoskeleton was visualized by taking 10^7 ^cells and resuspending them in 50 μl of 37% formaldehyde and incubating for 15 min at 25°C. Cells were collected and resuspended in a second fix solution (50 μl of 37% formaldehyde and 500 ml of PBS (pH 7.0) and incubated at room temperature for 1 h at 150 revolutions per minute. Cells were collected and washed three times in 100 μl of PBS. In the final wash cells were resuspended in 30 μl PBS and 8 μl of Alexa Fluor^® ^568 phalloidin dissolved in ethanol (Invitrogen-Molecular Probes A12380), which is a high affinity probe specific for F-actin. Cells were incubated at 4°C for 1 h in the dark and then washed with 50 μl of PBS and resuspended in a final volume of 50 μl PBS. The actin cytoskeleton was visualized using the TX RED HYQ fluorescent filter (532-587 nm) [51]. Actin cytoskeleton structures were classified as either normal or abnormal. Normal actin cytoskeleton contained tubules in the mother cell that extended to he bud where they terminated as circular stained patches. Abnormal actin cytoskeleton lacked tubules and contained large actin patches throughout the mother cell and bud. For each time point at least 100 cells were examined.

## Abbreviations

DCF: dichlorofluorescein diacetate; GFP: green fluorescent protein; NADPH: nicotinamide-adenine-dinucleotide phosphate (reduced form); PBS: phosphate buffered saline; PPP: pentose phosphate pathway; ROS: reactive oxygen species; SD: synthetic defines; SEM: scanning electron microscopy; TEM: transmission electron microscopy; YNB: yeast nitrogen base; YPD: yeast extract-peptone-dextrose.

## Competing interests

The authors declare that they have no competing interests.

## Authors' contributions

SAA performed the majority of the experiments including ROS analyses and mitochondria, vacuole and chromatin damage studies. SAA also assisted with analyses and manuscript writing. WC performed the actin staining and analysis of those data. JMM performed all of the TEM and assisted in the analysis of those data. ZC assisted with ROS and cell death experiments. AL assisted with image gathering and analysis. PJS and ZLL contributed to data analysis and manuscript revision. SWG directed the study and wrote the manuscript. All authors have read and approved the final manuscript.

## Supplementary Material

Additional file 1 **Figure S1 - Furfural causes exponentially growing yeast to enter a growth lag phase**. Exponentially growing yeast cells in synthetic complete medium were either untreated (circle) or treated with 25 m*M *(square) or 50 m*M *furfural (triangle) and allowed to continue to grow at 30°C. At the indicated time points aliquots of cells were removed and cell density measured (A_600_). Click here for file
