# Supplementary figures and images for: Furfural induces reactive oxygen species accumulation and cellular damage in Saccharomyces cerevisiae
Source: Biotechnol Biofuels. 2010 Jan 15;3:2. doi: 10.1186/1754-6834-3-2 (PMC2820483; doi:10.1186/1754-6834-3-2)

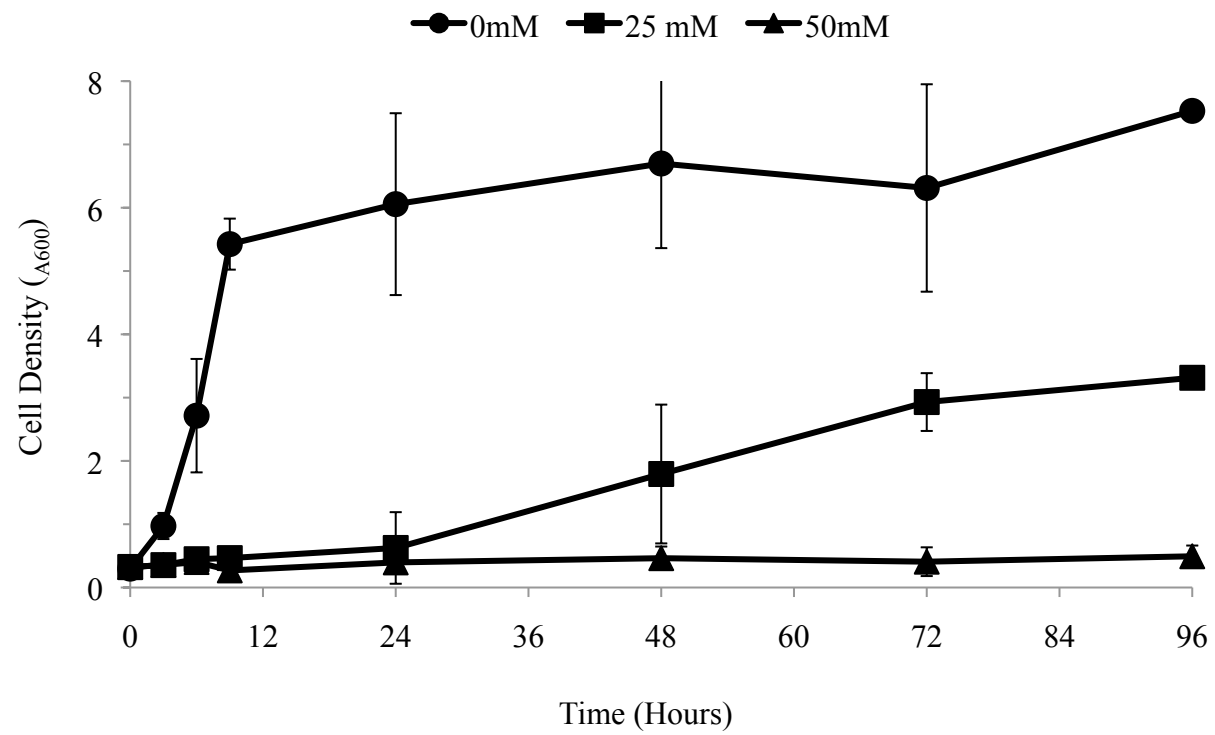

Supplement: Additional file 1 — Figure S1 - Furfural causes exponentially growing yeast to enter a growth lag phase. Exponentially growing yeast cells in synthetic complete medium were either untreated (circle) or treated with 25 mM (square) or 50 mM furfural (triangle) and allowed to continue to grow at 30°C. At the indicated time points aliquots of cells were removed and cell density measured (A600). [file 1754-6834-3-2-S1.PDF]
